# Management challenges in Heyde syndrome: A case report of recurrent gastrointestinal bleeding and aortic stenosis in an octogenarian

**DOI:** 10.21542/gcsp.2025.36

**Published:** 2025-08-30

**Authors:** Muhammad Salman Sabri, David Glass, Aheed Javaid, John Dudzinski

**Affiliations:** 1 Department of Internal Medicine, Jefferson Abington Hospital, Abington, PA, USA; 2 Department of Cardiology, Jefferson Abington Hospital, Abington, PA, USA

## Abstract

Heyde syndrome is defined by the triad of aortic stenosis, gastrointestinal bleeding from angiodysplasias, and acquired von Willebrand disease. Management of aortic stenosis in Heyde syndrome poses a unique clinical challenge, particularly in elderly patients with multiple comorbidities. We present the case of an 87-year-old woman with severe anaemia secondary to gastrointestinal bleeding, moderate-to-severe aortic stenosis, and confirmed acquired von Willebrand disease. Despite endoscopic intervention and medical management, the patient continued to experience recurrent bleeding and haemodynamic instability. Although aortic valve replacement is known to mitigate bleeding risk by correcting shear stress-induced von Willebrand factor abnormalities, the patient’s frailty, atrial fibrillation, and preference for conservative management complicated decision-making. This case highlights the complexities and therapeutic dilemmas in managing aortic stenosis in Heyde syndrome, particularly balancing the risks and benefits of surgical intervention versus conservative treatment in a vulnerable population.

## Introduction

Heyde syndrome (HS) is characterized by the triad of aortic stenosis (AS), gastrointestinal (GI) bleeding due to angiodysplasias, and acquired von Willebrand disease (VWD)^1^. In HS, GI bleeding is often associated with moderate-to-severe AS^1^, with symptoms frequently improving following aortic valve replacement (AVR)^6^. However, antithrombotic therapy remains necessary after AVR^6^. Comprehensive studies comparing conservative and surgical management of GI bleeding in HS patients, particularly among the octogenarian population, are lacking. This case addresses a critical gap in the literature surrounding the management of HS in elderly, frail patients who are not candidates for aortic valve replacement (AVR) or transcatheter aortic valve replacement (TAVR). While AVR is the definitive treatment for HS, there is limited evidence guiding conservative management in patients who decline intervention due to frailty, comorbidities, or personal preferences. Our case provides a detailed, multidisciplinary approach to managing HS in such a context, including the use of medical therapy (octreotide, iron supplementation), risk scoring, and integration of palliative care. This report highlights the real-world challenges of balancing disease modification with quality of life in a vulnerable population and underscores the need for individualized treatment planning.

## Case Presentation

**Figure 1.**
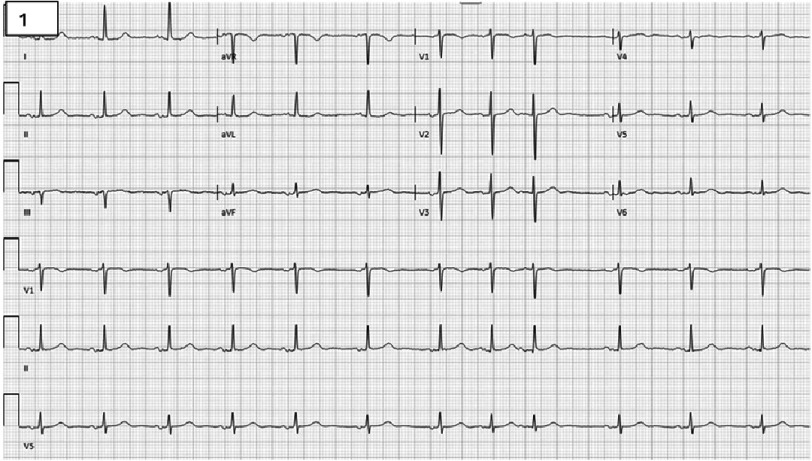
Electrocardiogram demonstrates normal sinus rhythm with premature supraventricular complex.

An 87-year-old woman with a medical history of type 2 diabetes mellitus, depression, hypertension, and hyperlipidemia presented to the hospital following outpatient laboratory results that revealed anemia, with a hemoglobin level of 6.8 g/dL. The patient was independent at home and able to carry out her activities of daily living, although she was not physically very active. She did not report any symptoms such as chest pain, shortness of breath, palpitations, dizziness, syncope, or leg swelling. Additionally, she had no recent history of requiring iron supplementation or blood transfusions. Physical examination was notable for conjunctival pallor and a crescendo-decrescendo murmur at the right upper sternal border radiating to the carotids; the chest was clear to auscultation, and the remainder of the examination was unremarkable. Laboratory evaluation showed a hemoglobin level of 6.6 g/dL, platelet count of 130,000 cells/μL, INR of 1.3, PTT of 45 s, and PT of 14 s. Electrocardiogram demonstrated normal sinus rhythm with premature supraventricular complexes (Figure 1).

**Figure 2.**
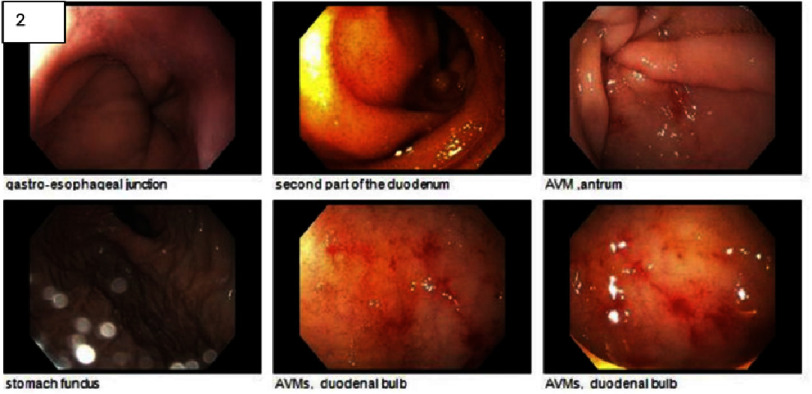
Endoscopy demonstrates arteriovenous malformations in the gastric antrum and distal duodenal bulb.

**Figure 3.**
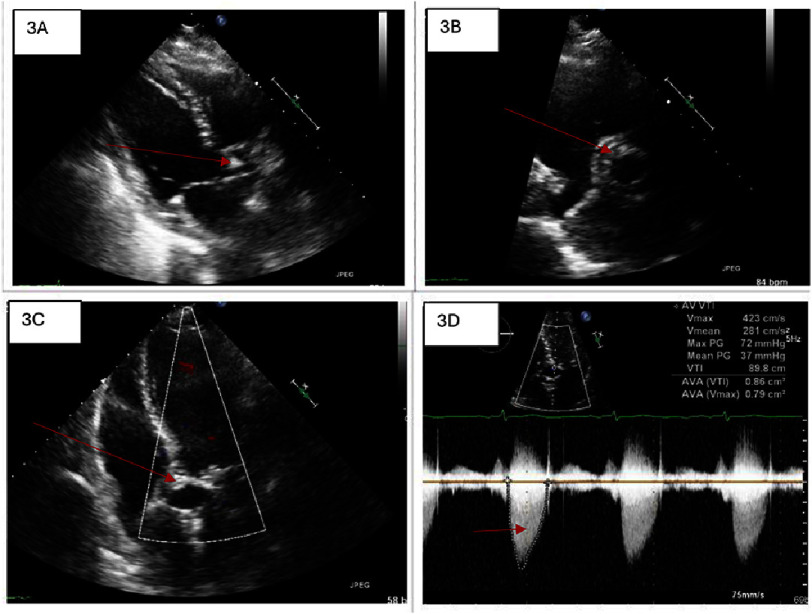
Transthoracic echocardiogram demonstrates a calcified and narrowed aortic valve as depicted by red arrows in 3A (Parasternal long axis), 3B (Parasternal short axis), and 3C (Apical five chamber view). The valve area was measured at 0.8 cm^2^ with a dimensionless index of 0.33 (Figure 3D).

Endoscopy (Figure 2) revealed prominent gastric antral and distal duodenal bulb angiovascular malformations (AVM) that were treated with thermal therapy. The colonoscopy was unremarkable. Transthoracic echocardiography (TTE) (Figure 3, Video S1) showed a left ventricular ejection fraction of 60–65%, normal left-sided filling pressures, and moderate-to-severe AS (mean gradient 36 mmHg, dimensionless index 0.33, and valve area 0.8 cm^2^). Cardiac catheterization revealed minimal-to-mild coronary artery irregularities. Von Willebrand factor (VWF) antigen levels were decreased with a positive ristocetin cofactor test, supporting the diagnosis of VWD. The patient also had low ferritin, normal vitamin B12 and folate levels, and an elevated reticulocyte count. She was transfused with blood, and hematology was consulted; octreotide was initiated for gastrointestinal bleeding.

Hematology also recommended addressing AS to improve the underlying acquired VWD. The patient experienced new-onset atrial fibrillation with a rapid ventricular rate ranging from 140 to 150 beats per minute. Management included cautious administration of intravenous fluids, blood transfusions, and metoprolol; however, her blood pressure remained on the lower end of normal with beta-blocker use. She was subsequently loaded with digoxin, which effectively reduced her heart rate to the 90 s. Due to ongoing gastrointestinal bleeding from arteriovenous malformations (AVMs) and low hemoglobin levels requiring blood transfusions, she was not considered a candidate for anticoagulation therapy.

The patient was evaluated by both cardiothoracic surgery and interventional cardiology. Her calculated European System for Cardiac Operative Risk Evaluation II (EuroSCORE II) was 4.95%, indicating moderate operative risk. Based on the Society of Thoracic Surgeons (STS) risk model for isolated aortic valve replacement, the estimated operative mortality was 13.7%, with an overall morbidity and mortality risk of 26.1%. Specific predicted perioperative complications included a 4.67% risk of stroke, 5.85% risk of renal failure, 6.92% risk of reoperation, and an 18.8% likelihood of requiring prolonged mechanical ventilation. The risk of deep sternal wound infection was minimal at 0.05%. Additionally, there was a 27.1% chance of a prolonged hospital stay exceeding 14 days, and a 9.33% likelihood of discharge within six days. Given the high surgical risk, transcatheter aortic valve replacement (TAVR) was recommended over open surgical aortic valve replacement.

The hematology team supported treatment of the aortic stenosis (AS) to address the underlying acquired von Willebrand disease (VWD), and in the interim recommended intravenous (IV) iron and an erythropoiesis-stimulating agent (ESA). They also discussed potential use of other agents such as lanreotide, which would only be available in the outpatient setting. Cardiology recommended computed tomography (CT) of the aorta with a TAVR planning protocol. After a comprehensive discussion, the patient declined all invasive and even minimally invasive interventions, choosing instead to allow for the natural progression of the disease in light of her frailty and personal values. Although the family initially disagreed with the patient’s decision, they ultimately supported her wishes following repeated affirmations of her choice. After discussion of both surgical and conservative management options for AS, the patient elected to pursue conservative care.

The patient was initially treated with intravenous octreotide, which was later transitioned to subcutaneous administration. This resulted in successful control of gastrointestinal bleeding and eliminated the need for additional blood transfusions. Her hemoglobin levels remained stable, consistently above 7 g/dL. The patient’s hemoglobin increased from 6.6 g/dL on admission to 8.6 g/dL at discharge following six units of packed red blood cell transfusions, four doses of intravenous ferric gluconate, and initiation of oral ferrous sulfate 325 mg daily. The patient was functionally independent prior to admission but experienced notable deconditioning during hospital stay, including urinary retention requiring Foley catheterization and prolonged nothing-by-mouth (NPO) status due to GI bleeding. Physical therapy assessed her with a Basic Mobility Score of 11 and recommended discharge to a skilled nursing facility. Patient and family requested a palliative care meeting and opted for home hospice care. The patient was discharged from the hospital to home hospice. No blood draws or laboratory tests were performed during hospice, in accordance with comfort-focused care. Anticoagulation and antiplatelet therapy were not initiated due to ongoing gastrointestinal bleeding, low hemoglobin, and the patient’s decision to pursue hospice care. This was a deliberate and permanent decision aligned with her palliative goals. The patient passed away 2 months after discharge.

## Discussion

HS typically occurs in patients aged 65 and older. Risk factors contributing to HS include female gender, coronary artery disease, heart failure, type 2 diabetes mellitus, chronic kidney disease, and atrial fibrillation^2^. In HS, acquired VWD is primarily attributed to the shearing of VWF multimers as they pass through the narrowed aortic valve. This process leads to the proteolysis of VWF multimers and disrupts platelet-mediated hemostasis. VWF multimers are known to contribute to the suppression of angiogenesis through integrin-mediated signaling and vascular endothelial growth factor signaling. Consequently, the reduction of VWF multimers in HS promotes increased angiogenesis, contributing to the development of angiodysplasia and GI bleeding^2–4^. Severe AS can lead to loss of pulsatility or reduced pulse pressure causing decreased GI perfusion. This subsequently leads to hypoxia-induced dilation of the blood vessels, inducing the development of angiodysplasias in the GI tract^5^.

Patients with HS may present with symptoms such as hematochezia, melena, abdominal pain, pallor, exertional dyspnea, chest pain, presyncope, and syncope, often related to AS and concurrent anemia due to VWD. Patients can also present with low outpatient hemoglobin in serial laboratory tests with absence of symptoms, especially elderly patients who are not mobile at baseline, as seen in our case. Physical examination findings may include a crescendo-decrescendo murmur at the right upper sternal border, pallor, tachycardia, and petechiae. Laboratory investigations typically reveal anemia. While von Willebrand factor (VWF) antigen and ristocetin cofactor activities are usually normal, a prolonged partial thromboplastin time (PTT) may be present due to decreased factor VIII levels secondary to acquired VWD. Confirmation of reduced levels of large high-molecular-weight (HMW) multimers of VWF can be achieved through VWF multimer assay using gel electrophoresis as a confirmatory test. An echocardiogram is recommended to assess the severity of aortic valve disease.

Aortic valve replacement is the definitive management of HS^1,8^. Elderly patients, such as the one in our case, are generally not candidates for aortic valve repair due to the high risk of operative mortality and morbidity, as well as individual and family preferences. Initial management of Heyde syndrome (HS) in this population typically includes transfusion of blood products and discontinuation of anticoagulants, antiplatelet agents, nonsteroidal anti-inflammatory drugs (NSAIDs), and corticosteroids. Other conservative approaches include strategies such as iron infusions and the use of octreotide. Endoscopic interventions are also employed to achieve hemostasis and manage GI bleeding. In cases of recurrent and uncontrollable GI bleeding, AVR serves as the definitive treatment for AS^8^, resulting in improvements in VWF levels and resolution of GI bleeding. AVR has demonstrated efficacy in halting bleeding in 93% of patients with HS^1^. When selecting a valve type, bioprosthetic valves are often preferred over mechanical valves to reduce the risk of recurrent GI bleeding necessitating anticoagulation. The choice between TAVR and SAVR depends on individual patient comorbidities, with both procedures showing similar efficacy. TAVR is associated with fewer perioperative complications such as myocardial infarction and stroke, and reduced blood transfusions. In general, TAVR is gaining popularity as an alternative to surgery for treating valvular heart disease. Concerns persist over significant bleeding and thromboembolic complications following AVR, highlighting the importance of minimizing the use of anticoagulants and antiplatelet agents in patients with suspected or confirmed Heyde syndrome.

Conservative measures may temporize bleeding and anemia, but do not resolve the underlying cause. Most cases managed with endoscopic interventions eventually relapse and bleeding recurs unless the valve pathology is corrected. There is evidence supporting the use of systemic bevacizumab for managing refractory GI bleeding and thus facilitating subsequent aortic valve replacement in these challenging cases^7^. While our patient was managed conservatively with blood transfusions, other interventions such as endoscopy or systemic bevacizumab could not be done due to limited evidence showing efficacy for endoscopy to manage bleeding and non-availability of lanreotide and bevacizumab while inpatient.

In summary, the management of elderly patients with Heyde syndrome (HS) should begin with a conservative approach, particularly when they are not surgical or transcatheter aortic valve replacement (TAVR) candidates. It is essential to involve the family and palliative care team early in the decision-making process to ensure that the patient’s values, beliefs, and goals of care are fully understood and respected. A thorough discussion should be held regarding the risks and potential benefits of aortic valve replacement, including symptom relief and possible improvements in morbidity and mortality, compared to conservative management. However, as in our case, the patient was asymptomatic as an outpatient and expressed a strong desire to avoid invasive procedures. Despite experiencing worsening frailty during hospitalization, she remained consistent in her preference for nonintervention. This highlights the importance of individualized care planning and respecting patient autonomy, especially in the context of advanced age, frailty, and complex comorbidities.

## Conclusion

Managing AS in the context of HS is particularly challenging, especially in elderly patients with significant comorbidities. While AVR can address the underlying pathophysiology and reduce bleeding episodes, the decision must weigh the risks of surgery against the patient’s overall frailty, bleeding risk, and personal preferences. This case highlights the complexities of managing Heyde syndrome in elderly, frail patients who declined aortic valve intervention. Although medical therapy including octreotide, blood transfusions, and iron supplementation initially stabilized her gastrointestinal bleeding, she experienced progressive functional decline and ultimately opted for home hospice care. Her hemoglobin improved from 6.6 to 8.6 g/dL with six units of packed red blood cells and four doses of intravenous ferric gluconate, followed by oral ferrous sulfate. Despite a Basic Mobility Score of 11 and recommendation for skilled nursing facility placement, the patient prioritized quality of life and chose hospice. Anticoagulation and antiplatelet therapy were withheld due to high bleeding risk and the patient’s goals of care. This case underscores the importance of shared decision-making, multidisciplinary coordination, and early palliative care involvement, especially in managing HS in elderly patients who prefer conservative treatment over procedural intervention.

## Disclosures

The authors have nothing to disclose.

## Funding

The authors received no funding to disclose.

## Patient consent

Informed consent for publication of this case and associated clinical details was obtained from the patient’s family. The family reviewed the case information and expressed no objections to its use for educational and academic purposes.
